# Biological Surgical Options in Young Patients for the Treatment of Severe Aortic Stenosis: Is the Jury Still Out? A Review

**DOI:** 10.31083/j.rcm2308274

**Published:** 2022-07-26

**Authors:** Khalil Khalil, Jessica Forcillo

**Affiliations:** ^1^Department of Cardiac Surgery, Centre Hospitalier de l’université de Montréal (CHUM), Montreal, QC H2X 0C1, Canada; ^2^Department of Surgery, Faculty of Medicine, Université de Montréal, Montreal, QC H3T 1J4, Canada

**Keywords:** aortic stenosis, young adults, aortic valve replacement, bioprosthetic valve, mechanical valve, ross procedure

## Abstract

Aortic interventions remain the most effective treatment for severe aortic stenosis. In the recent years, advances in bioprosthetics and newer data have reduced the cut-off age for the use of bioprosthetic valves in younger patients, but the debate on whether to favor mechanical valves in younger patients remains a constant, especially with the undesired effects and considerations of anticoagulation therapy with vitamin K antagonists in this age group. Other options like the Ross procedure are gaining traction, despite still being undervalued and necessitating expertise centers. Hemodynamic considerations and durability of these options are important to consider, especially in this age group. Regardless of the choice of the prosthesis, patient informed consent is paramount since the decision affects the lifetime management of their initial condition, and expectations given must remain realistic.

## 1. Introduction

Aortic stenosis (AS) remains the most common type of valvular heart disease in Western countries and can affect patients of any age. Data of prevalence of AS in the general population is lacking but it is estimated at 2–4% in patients ≥75 years of age and at 1% in patients <55 years old, with stenotic bicuspid valve being the second most common etiology [1, 2, 3]. In the absence of treatment and after the onset of symptoms, severe AS has a mortality of nearly 25% per year [4]. With the lack of definitive pharmacological therapy to alter the natural history of severe symptomatic aortic stenosis [4], direct intervention on the aortic valve remains the most effective treatment to relieve the left ventricular outflow obstruction. This can be achieved either surgically with the replacement of the aortic valve, or percutaneously via transcatheter aortic valve replacement (TAVR). Several valve options for surgical aortic valve replacement (SAVR) are available and include mechanical valves, bioprosthetic valves, aortic homografts, and pulmonary autografts (Ross procedure). Every valve option has its own specificities and implications on long-term outcomes and thus the choice should be tailored to every patient individually. In this article, we will focus on the biological surgical options in young patients for the treatment of severe aortic stenosis.

## 2. Changes in Recommendations

**Fig. 1.**
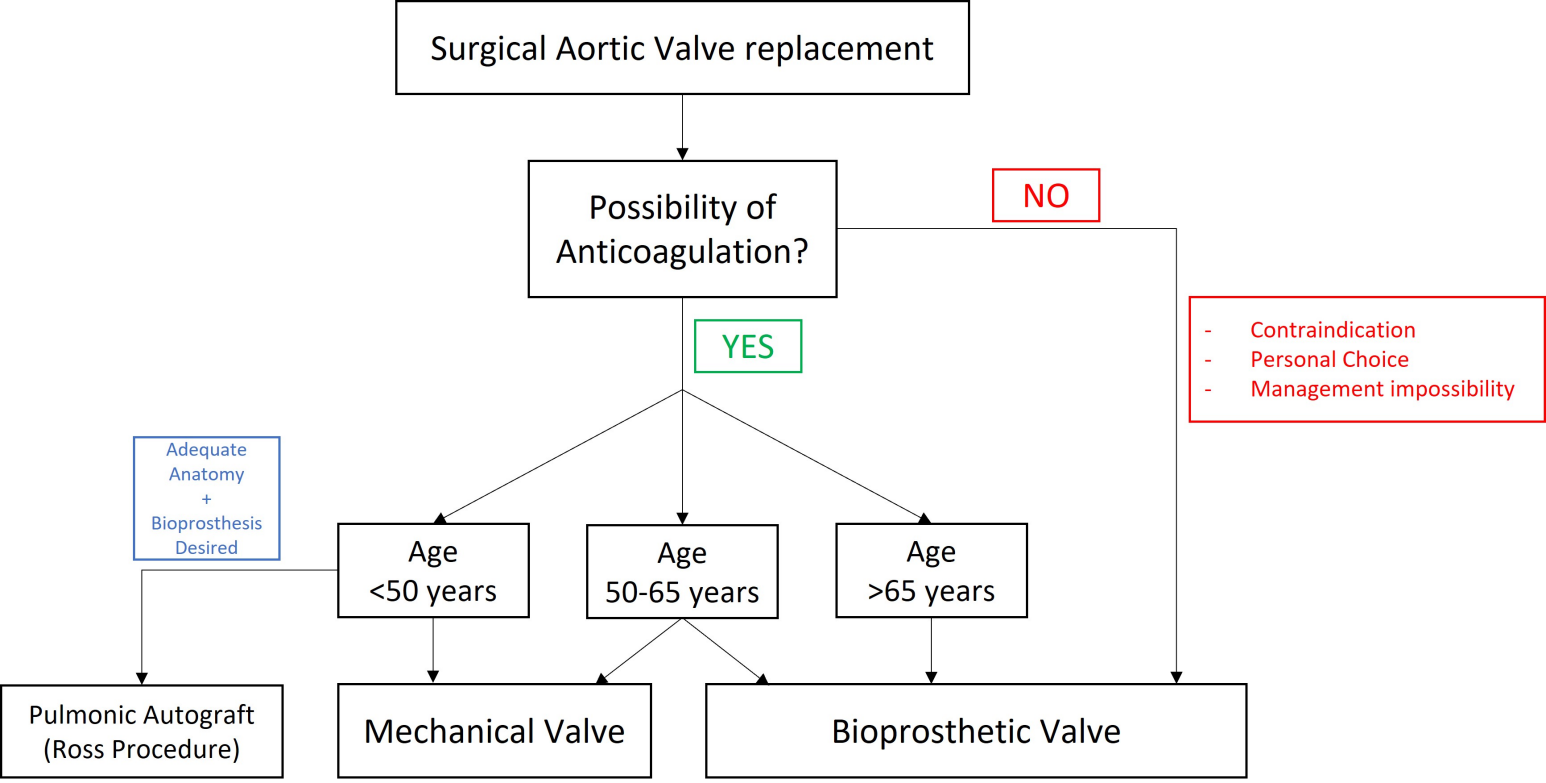
**Choice process of prosthetic valve adapted from the 2020 ACC/AHA Guideline for the Management of Patients With valvular Heart Disease [8]**.

The previous guidelines for the management of valvular heart disease from the American College of Cardiology (ACC)/American Heart Association (AHA) (2014) [5] as well as that from the European Society of Cardiology (ESC) and the European Association for Cardio-Thoracic Surgery (EACTS) (2017) [6] recommended, in terms of the choice of the prosthesis, the use of mechanical valves in patients <60 years of age for both guidelines and the use of bioprosthetic valves in patients >65 years of age for the Europeans or >70 years of age for the Americans, with a grey zone between the ages of 60 and 65–70. These IIa recommendations were based on their publication on long-term results of the Veterans Affairs landmark randomized trial on heart valve replacement with mechanical vs biological valves [7]. However, since then, the advancements in valve designs and production and the increase in publications and newer data regarding the bioprosthetic valves have led to change the recommendations in the newer versions of the guidelines. In fact, the 2020 ACC/AHA guideline now recommends the use of a mechanical valve in patients <50 years of age and a bioprosthetic valve in patients >65 years of age [8], while the 2021 ESC/EACTS guideline did not change in that regard [9]. The guidelines mention that in case a patient <50 years of age desires a bioprosthesis and that their anatomy permits, a pulmonic autograft can be used and a Ross procedure can be performed [8]. The choice process of prosthetic valve depending on age based on the 2020 ACC/AHA guideline is shown in Fig. 1 (Ref. [8]).

## 3. The Bioprosthetic Valve Choice

**Fig. 2.**
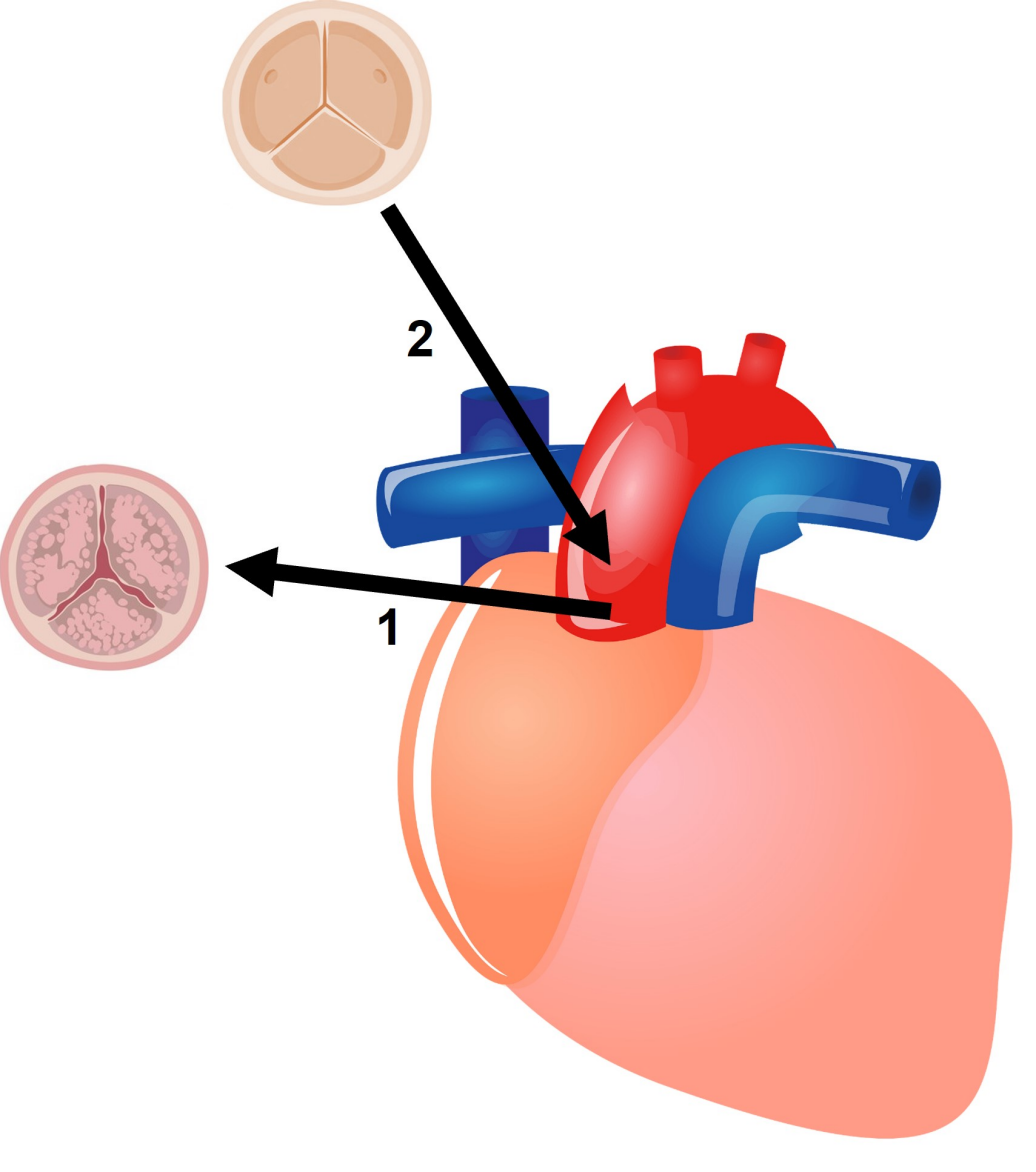
**Schematic representation of a biological surgical aortic valve replacement**. (1) The diseased aortic valve is removed. (2) A bioprosthesis is inserted in its place.

Throughout the recent years, the advancements in valve designs and durability allowed for a dramatic increase in the number of bioprosthetic aortic valve implants in comparison to mechanical one. In fact, from 1997 to 2012, in the state of New York alone, the use of bioprosthetic valves went from 15 to 74% in young adults (age 50–69 years old) [10]. In the German Heart Surgery Report of 2020, 88% of patients had a bioprosthetic valve implanted [11]. The schematic representation of a bioprosthetic surgical aortic valve replacement is shown in Fig. 2. Guidelines have also further decreased the age cut-off for the use of biological prosthesis during aortic valve replacement throughout the recent years [8, 9]. This can be explained by the advancements in design and the long-term data on durability and survival of patients who had those prostheses implanted, which are encouraging [12]. Other factors that contributed to the potential use of bioprosthetics in younger patients are the advents of new technologies that changed our field such as TAVR, and the advancements in that regard that allowed for more reliable implants and the possibility for Valve-in-Valve procedure in the future. In addition, regardless of durability and advancements, bioprosthetics have been used in young patients who refuse (or are contraindicated) to take long-term anticoagulation [10, 11]. According to Dr. Bourguignon and his colleagues, the expected valve durability of the Carpentier-Edwards Perimount aortic valve was 17.6 years for the younger patients (<60 years of age) [13] and 22.1 years for patients between 60–70 years of age [14]. They found out that the valve-related actuarial survival rate in the <60 years of age group was 93.7% ± 1.5% at 10 years, 86.5% ± 2.8% at 15 years, and 83.6% ± 3.4% at 20 years [13]. In the same age group, actuarial freedom of structural valve deterioration (SVD) at 10, 15, and 20 years was 86.8% ± 2.5%, 66.8% ± 4.2%, and 37.2% ± 5.4% respectively, and freedom from reoperation due to SVD was 88.3% ± 2.4%, 70.8% ± 4.1%, and 38.1% ± 5.6% respectively [13, 14]. The 5 years data of the COMMENCE trial [15] (Carpentier-Edwards Inspiris valve) and the PERIGON trial [16] (Medtronic Avalus valve) are also encouraging showing no structural valve deterioration at 5 years with these new generation bioprosthetic aortic valves. With the lowered risk of reoperation, and the reduced morbidity and mortality related to it [12], increasing reported evidence is suggesting the use of these valves in patients even younger than 50 years of age. What would this mean for the patients, down the line, in terms of survival and subsequent procedures?

## 4. The Debate with the Mechanical Valve

Despite the advancements in bioprosthetic valves, the debate regarding whether mechanical or bioprosthetic valves should be used in patients aged 50–70 years remains a constant. Leviner and colleagues published in 2022 a meta-analysis comparing mechanical vs bioprosthetic aortic valve replacement in patients younger than 70 years old. They showed an overall survival benefit for patients who received a mechanical valve [17]. Also, in 2017, Goldstone *et al*. [18] published a comparative study comparing both types of valves. They showed that among patients who underwent aortic valve replacement, receipt of a biologic prosthesis was associated with significantly higher 15-year mortality than receipt of a mechanical prosthesis among patients 45 to 54 years of age (30.6% vs 26.4% at 15 years; *p* = 0.03) but not among patients 55 to 64 years of age.

On the counter part, Joanna Chikwe and her group compared mortality and morbidity in young adults (18–50 years of age) in the states California and New York who received mechanical versus tissue valve between 1997 and 2006. They observed that the use of bioprosthetic valves increased from 14% to 47% from 1997 to 2014. There was no survival difference with bioprosthetic versus mechanical aortic valves in the propensity score-matched cohort: actuarial 15-year survival was 79.0% vs 81.5 respectively; *p* = 0.20). There was more stroke and bleeding in the mechanical valve group and more reoperation in the bioprosthesis valve group. They suggested that in patients 18–50 years, bioprosthesis are a reasonable alternative to mechanical valves for aortic valve replacement [19]. Also, two other propensity-matched analyses found that survival was comparable between the types of valves. McClure *et al*. [20] reported a single-center analysis of 722 propensity-matched patients younger than 65 years and a mean age of 53 who were followed for a median of 6–7 years. Survival after bioprosthetic and mechanical implantation was 78% vs 79% at 10 years, respectively, and 65% vs 75% at 15 years, respectively (*p* = 0.75). Chiang *et al*. [10] analyzed 2002 patients aged 50–69 years from the New York State registry and followed these patients for a median of 10.8 years. At 15 years, survival was 60.6% in the bioprosthetic valve group and 62.1% in the mechanical group (*p* = 0.74). A group from Germany published in 2021 a propensity-adjusted analysis in patients of two subgroups (<60 and >60 years of age) who had either a biological or a mechanical valve. They found that the long-term survival at 10 years, after surgical aortic valve replacement regardless of age, was similar in patients with mechanical and in patients with biological implants (69.8% vs 79.1%, *p* = 0.83). The same study also showed no benefits of the mechanical prosthetics over the bioprosthetics regarding cumulative major adverse cardiovascular and cerebral events rates in patients <60 years of age (4.6% vs 7.3%, *p* = 0.83) [21].

Anticoagulation therapy with vitamin K antagonist (VKA) in the context of a mechanical prosthetic remains necessary to prevent thrombo-embolic and valve thrombosis events as newer anticoagulants have noy yet been proven to be safe or effective in these patients. This corresponds to a level I recommendation in the current guidelines. These same guidelines only find it reasonable to give aspirin 70 to 100 mg daily post-bioprosthetic AVR in all patients and VKA for 3 to 6 months in patients who are at low risk of bleeding. However, these correspond to a level IIa recommendation [8]. The prospective randomized On-X valve anticoagulation clinical trial (PROACT) showed that a lower INR target of 1.5 to 2 post-operatively, with the On-X mechanical prosthesis, decreases the incidence of both major and minor bleeding events when compared to the control group with an INR of 2 to 3 (1.48%/pt-yr versus 3.31%/pt-yr, and 1.18%/pt-yr versus 3.31%/pt-yr respectively) without increasing the risk of thrombo-embolic events (2.96%/pt-yr versus 1.85%/pt-yr, *p* = 0.178) [22, 23]. The LOWERING-IT trial evaluated the impact of lower anticoagulation targets (INR = 1.5–2.5 vs 2–3) with various mechanical valves and showed similar results to the PROACT trial with a significant decrease in bleeding (OR = 0.36, CI: 0.11–0.99, *p* = 0.04) and no difference in thrombo-embolic events (OR = 0.33, CI: 0.006–4.20, *p* = 0.6) [23, 24]. Despite the lower dosages, undesired effects and restrictions related to long-term VKA treatment sometimes push patients to avoid mechanical prosthesis because of medication interactions, dietary restrictions, the inconvenience of monitoring, and the need to restrict participation in certain activities, especially in young patients [8]. The management of VKA during pregnancy is also a concern in women of childbearing age undergoing an AVR [25, 26, 27]. Is the jury still out for the use of mechanical or biological valves in those younger patients?

## 5. The Ross Procedure 

**Fig. 3.**
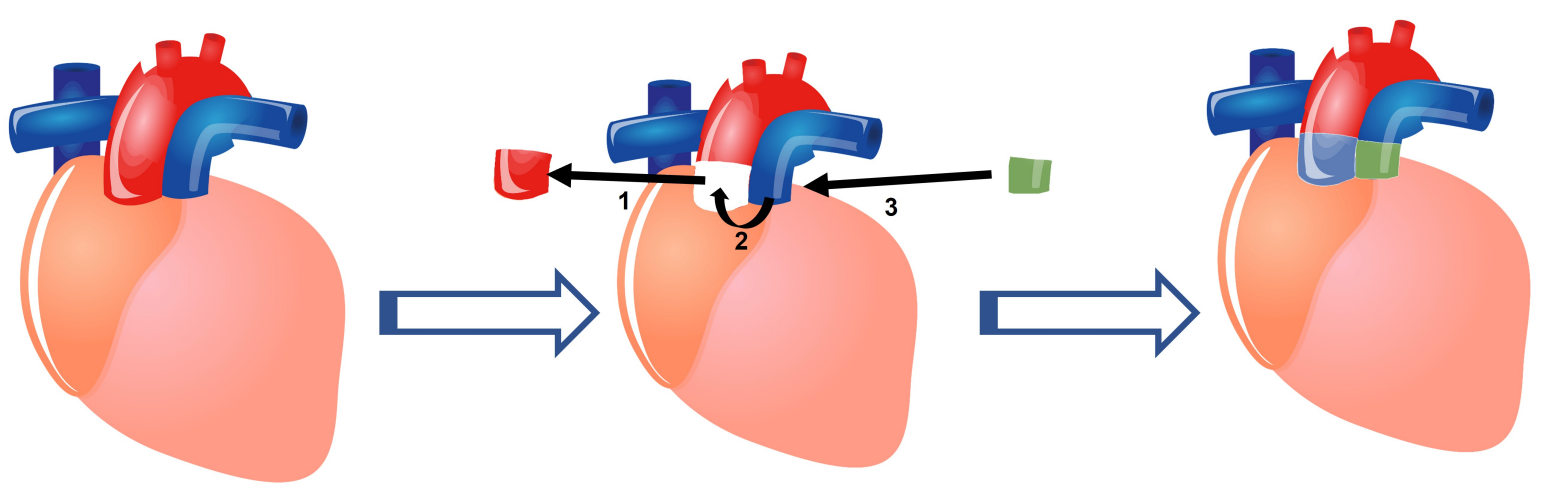
**Schematic representation of a Ross procedure (Pulmonary Autograft)**. (1) The diseased aortic valve is removed with a portion of the aorta. (2) The patient’s own pulmonic valve and a portion of the pulmonary artery are excised and placed in the aortic position. (3) A homograft (Allograft) consisting of the pulmonary valve and a portion of the pulmonary artery are placed in the pulmonary position.

Other options do exist, like the pulmonary autograft (Ross procedure). The schematic representation of a Ross procedure is shown in Fig. 3. The current guideline mentions the possibility of choosing the pulmonary autografts in young patients <50 years of age if they desire a bioprosthetic option instead of the mechanical one and have an adequate anatomy to perform the Ross procedure [8], as seen in Fig. 1. We can argue that these other options have been undervalued, and this is demonstrated in a query of the Society of Thoracic Surgeons database [28]. Of 2180 patients 18 to 30 years of age listed in the adult Society of Thoracic Surgeons database who received an aortic valve replacement between 2008 and 2011, 9% had valve repairs, 2% had the Ross operation, and 85% had a prosthetic valve. Dr. El-Hamamsy and his team recently published a study comparing the Ross procedure vs, biological and mechanical aortic valve replacement in adults (18–50 years) undergoing aortic valve surgery. They show that in young adults, the Ross procedure is associated with better long-term survival and freedom from valve-related complications compared with prosthetic AVR. Also, at 15 years, actual survival after the Ross procedure was 93.1% (95% CI: 89.1%–95.7%), similar to that of the age-, sex-, and race-matched U.S. general population. It was significantly lower after biological AVR (HR: 0.42; 95% CI: 0.23–0.075; *p* = 0.003) and mechanical AVR (HR: 0.45; 95% CI: 0.26–0.79; *p* = 0.006) [29]. Other studies have also reported excellent long term survival data similar to that of the general population (age- and sex-matched), unlike prosthetics, mechanical or biological, that cannot restore normal life expectancy in young patients undergoing an aortic valve replacement [13, 18, 30, 31, 32]. These contemporary studies have shown excellent survival even at 20+ years after the Ross procedure. David and colleagues reported a survival rate at 10, 15, and 20 years of 98%, 94%, and 94% respectively and a 10-year freedom from intervention of 97% for the autograft (AG) and 98% for the homograft (HG), a 15-year freedom from reintervention of 93% AG and 96% HG, and a 20-year freedom from intervention of 82% AG and 93% HG [33]. Skillington and colleagues also reported good survival rates at 10, 15, and 20 years of 98%, 97%, and 97% [34] while Martin and colleagues reported 94%, 92% and 84% respectively [35].

Despite the abundance of evidence, including randomized trial [19], a systematic review and meta-analysis [30] and several cohort studies, the use of the Ross procedure remains low, representing <1% of all AVRs in the STS database [36, 37]. Nevertheless, it has been shown that in dedicated aortic centers, despite the learning curve effect, operative outcomes are similar between the two approaches [38]. The recent 2020 ACC/AHA guidelines recommend the Ross procedure as class 2b recommendation for younger patients in centers of expertise [8].

## 6. Aortic Homograft

**Fig. 4.**
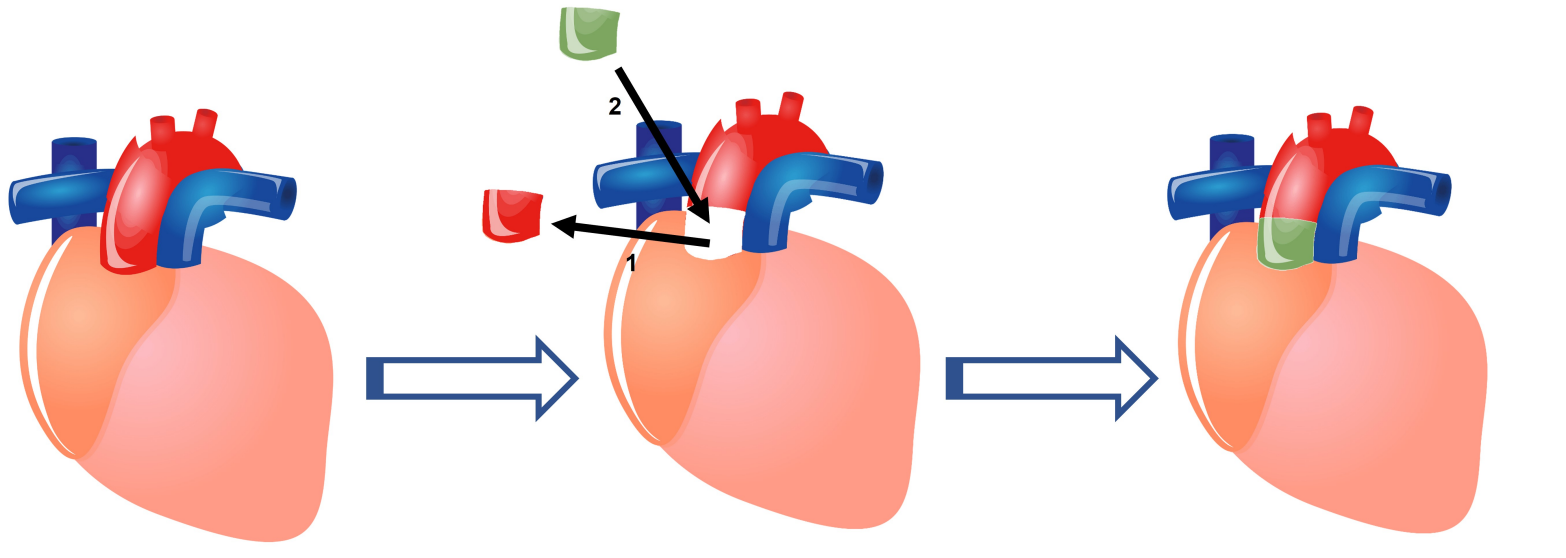
**Schematic representation of an aortic homograft procedure**. (1) The diseased aortic valve is removed with a portion of the aorta. (2) A homograft (Allograft) consisting of the aortic valve and a portion of the aorta are placed in its place.

Aortic homografts could also be an option for young patients, but it was shown that the survival of patients who received a homograft is decreased compared to patients who had the Ross procedure (at 13 years, survival was 78% ± 5% compared to 95% ± 3%) [30]. They have also been shown to have higher rates of SVD than bioprosthesis at 10 years (38% vs 19%) and at 20 years (82% vs 69%) [30, 39, 40]. Therefore, long-term results in the literature about the use of a homograft in patients without endocarditis are scarce and limited. Aortic homografts are rarely used in the context of AS nowadays and are mainly used for cases of endocarditis in which the avoidance of prosthetics and foreign objects in an infected area can be of advantage. The schematic representation of an aortic homograft procedure is shown in Fig. 4.

## 7. Hemodynamic Considerations for the Young Adults

Many young adults wish to maintain an active lifestyle and pursue higher levels of physical activities post-op. Therefore, the choice of procedure in young patient adds an extra factor that should be taken into consideration in addition to minimizing the risk of valve-related complication and restoring normal survival; it should also provide durable hemodynamic properties [41]. Both biological and mechanical prosthetics fix the annulus but are inherently obstructive. The pulmonary autograft, on the other hand, preserves aortic root mobility. This could be explained by the viability of the autograft and its capacity to remodel in the new hemodynamic environment. This allows for a better hemodynamic profile in patients who underwent the Ross procedure when compared to a prosthetic, whether mechanical or biological. The aortic gradient is a good indicator of hemodynamic performance. Lower gradients, closer to that of a normally functioning valve, are important at rest for any patient for congestive heart failure risk reduction [42]. A systematic review and meta-analysis by Um and colleagues reported that in observational studies, the mean aortic gradients were significantly lower at both discharge (–9 mmHg, CI: –13 to –5, *p *< 0.0001) and latest follow-up (–5 mmHg, CI: –7 to –3, *p *< 0.0001) in patients who underwent the Ross procedure [43]. Ross procedure was also associated with a lower mean aortic gradient at follow-up after 13 years in a randomized controlled trial when compared to aortic homograft (5 mmHg versus 30 mmHg) [30]. Pulmonary autograft has been shown in several studies to mirror the native healthy aortic valve in hemodynamic performance during activity by maintain a low gradient with maximal exercise [44, 45, 46]. Newer bioprosthetic valves have modified designs that allow the placement of a larger valve, that sits in the supra-annular position, while avoiding a high aortic gradient and decreasing the incidence of patient-prosthesis mismatch [47, 48]. With the younger age group of patients, exercise and active lifestyles are important factors to be taken into consideration and therefore hemodynamic performance during exercise is a sought benefit.

## 8. Conclusions

In conclusion, the use of a bioprosthetic valve implanted in the aortic position is increasing, but the choice of an aortic valve prosthesis is still a complicated matter, especially in young patients. Regardless of the decision, informed consent remains paramount since patients need to be carefully informed of the next steps, because this procedure becomes a lifetime management of their initial condition. This is why patient preference, in terms of valve type and willingness/ability to take anticoagulant therapy, is an important decisive factor that is integral to the decision process and that is clearly accounted for in the guidelines nowadays. With newer data coming every day, guidelines can change, and recommendations can be updated. Would the use of the novel anticoagulants with mechanical valve change the trends of implantation in those younger patients? And what about the Ross procedure? It seems to be a great operation in terms of survival, freedom from valve-related complications, and hemodynamic profile for those younger patients in dedicated centers of expertise. Will the number of cases increase in light of the most recent data, and will the Ross procedure finally penetrate the modern surgical practice? Other innovative methods like the AV-Neo, consisting of constructing leaflets from the patient’s own pericardium, are gaining traction worldwide with satisfactory results and are also potential areas of interest for future treatment options [49]. Those are the contemporary options that we have in our armamentarium to treat aortic valve stenosis and no matter the technique used, younger patients must be given very realistic expectations of the need of re-interventions, their survival benefit, and implications of having this or that type of procedure, and at what time point during their lifetime.
